# A retrospective investigation of spatial clusters and determinants of diabetes prevalence: scan statistics and geographically weighted regression modeling approaches

**DOI:** 10.7717/peerj.15107

**Published:** 2023-04-20

**Authors:** Jennifer Lord, Shamarial Roberson, Agricola Odoi

**Affiliations:** 1 Biomedical and Diagnostic Sciences, University of Tennessee, Knoxville, United States of America; 2 Florida Department of Health, Tallahassee, United States of America

**Keywords:** Spatial cluster detection, Diabetes prevalence, Spatial scan statistics, Geographically weighted regression, GWR, Determinants, Risk factors, GIS, Geographic Information Systems

## Abstract

**Background:**

Diabetes and its complications represent a significant public health burden in the United States. Some communities have disproportionately high risks of the disease. Identification of these disparities is critical for guiding policy and control efforts to reduce/eliminate the inequities and improve population health. Thus, the objectives of this study were to investigate geographic high-prevalence clusters, temporal changes, and predictors of diabetes prevalence in Florida.

**Methods:**

Behavioral Risk Factor Surveillance System data for 2013 and 2016 were provided by the Florida Department of Health. Tests for equality of proportions were used to identify counties with significant changes in the prevalence of diabetes between 2013 and 2016. The Simes method was used to adjust for multiple comparisons. Significant spatial clusters of counties with high diabetes prevalence were identified using Tango’s flexible spatial scan statistic. A global multivariable regression model was fit to identify predictors of diabetes prevalence. A geographically weighted regression model was fit to assess for spatial non-stationarity of the regression coefficients and fit a local model.

**Results:**

There was a small but significant increase in the prevalence of diabetes in Florida (10.1% in 2013 to 10.4% in 2016), and statistically significant increases in prevalence occurred in 61% (41/67) of counties in the state. Significant, high-prevalence clusters of diabetes were identified. Counties with a high burden of the condition tended to have high proportions of the population that were non-Hispanic Black, had limited access to healthy foods, were unemployed, physically inactive, and had arthritis. Significant non-stationarity of regression coefficients was observed for the following variables: proportion of the population physically inactive, proportion with limited access to healthy foods, proportion unemployed, and proportion with arthritis. However, density of fitness and recreational facilities had a confounding effect on the association between diabetes prevalence and levels of unemployment, physical inactivity, and arthritis. Inclusion of this variable decreased the strength of these relationships in the global model, and reduced the number of counties with statistically significant associations in the local model.

**Conclusions:**

The persistent geographic disparities of diabetes prevalence and temporal increases identified in this study are concerning. There is evidence that the impacts of the determinants on diabetes risk vary by geographical location. This implies that a one-size-fits-all approach to disease control/prevention would be inadequate to curb the problem. Therefore, health programs will need to use evidence-based approaches to guide health programs and resource allocation to reduce disparities and improve population health.

## Introduction

Diabetes mellitus and its complications represent an ongoing public health challenge in the United States (US). An estimated 26.9 million people in the US have been diagnosed with diabetes, while an additional 7.3 million who are estimated to be living with the condition have yet to be diagnosed (Centers for Disease Control and Prevention, 2020). Obesity, physical inactivity, and dietary pattern are among the most well-described modifiable risk factors of Type 2 diabetes mellitus (Bellou et al., 2018). Complications associated with chronic diabetes include cardiovascular disease, retinopathy, renal disease, neuropathy, and periodontal disease (International Diabetes Federation, 2017; Mealey & Ocampo, 2007). In 2017, diabetes had an age-adjusted mortality risk of 21.5 per 100,000 persons, making it the seventh leading cause of death in the US, and representing a 2.4% increase from the previous year (Kochanek et al., 2019). The condition accounts for a significant portion of annual healthcare spending in the US, as well as economic costs due to lost productivity. The total cost associated with diabetes in the US in 2017 was an estimated $327 billion, representing a 27% increase from 2012 (American Diabetes Association, 2018).

The burden of diabetes is not uniformly distributed across the US. In particular, the Southeastern US was characterized as the “diabetes belt” following the identification of spatial clusters of high diabetes risk in this region in analyses using 2007 and 2008 data (Barker et al., 2011; Shrestha et al., 2012). Rigorous statistical and epidemiological investigations of spatial patterns and identification of high-risk clusters are essential to expand upon the findings of previous, nation-wide studies as well as to overcome limitations associated with more descriptive investigations. For instance, the “diabetes belt” was defined using a prevalence cut-off of ≥11% (Barker et al., 2011). Moran Local Indicators of Spatial Association (LISA), which was used to identify a significant spatial cluster in this region in a subsequent study, has inherent limitations of multiple comparisons (Shrestha et al., 2012). Therefore, it is important to continue to monitor the spatial and temporal patterns of this condition using rigorous epidemiological approaches to better inform control and prevention efforts.

The statewide age-adjusted prevalence of diabetes in Florida has been higher than that of the nation overall during every year since 2011 (Centers for Disease Control and Prevention Division of Diabetes Translation, 2022). In addition, counties of northern Florida have been included in the southern extent of the large “diabetes belt” cluster in nation-wide studies (Shrestha et al., 2012). Our previous analysis focused specifically on identifying pre-diabetes and diabetes hotspots at the county level within Florida, using data from the 2013 Florida Behavioral Risk Factor Surveillance System (BRFSS). Multiple clusters with disproportionately high risks of the conditions were detected within the state, and individual-level determinants of diabetes status differed for cluster and non-cluster residents (Lord, Roberson & Odoi, 2020). These findings suggest that thorough, county-level investigations are valuable to inform targeted, evidence-based health planning. Furthermore, a follow-up analysis identified an increase in pre-diabetes prevalence from 8.0% to 10.5% as well as changes in high-risk cluster locations between 2013 and 2016, in addition to identifying significant predictors of the observed spatial patterns (Lord, Roberson & Odoi, 2021). Since the locations of high-risk clusters of pre-diabetes and diabetes may not be identical (Lord, Roberson & Odoi, 2020), ongoing surveillance is warranted for diabetes to enable periodic reassessment of spatial patterns and to identify any changes in these patterns over time. Similarly, since determinants of pre-diabetes and diabetes may differ, at least at the individual level (Lord, Roberson & Odoi, 2020), an ecological investigation to identify determinants of spatial patterns of diabetes prevalence is also warranted in order to guide population-level intervention strategies. Furthermore, determining whether the strength of associations between diabetes prevalence and these predictors varies based on location can help tailor such strategies to better meet the needs of communities. Findings from these investigations will provide critical information for evidence-based health planning, resource allocation and policy. Therefore, the objectives of this study were to identify: (1) spatial patterns and high-prevalence county-level diabetes clusters in Florida in 2016, (2) determinants of diabetes prevalence at the county level using global and local models, and (3) significant temporal changes in diabetes prevalence and spatial distribution between 2013 and 2016.

## Materials & Methods

### Ethics approval

This study was approved by the University of Tennessee, Knoxville Institutional Review Board (Number: UTK IRB-19-05440-XM), which determined that it was eligible for exempt review under 45 CFR 46.101. Category 4: Secondary research for which consent is not required. All methods were carried out in accordance with relevant guidelines and regulations.

### Study area

**Figure 1:**
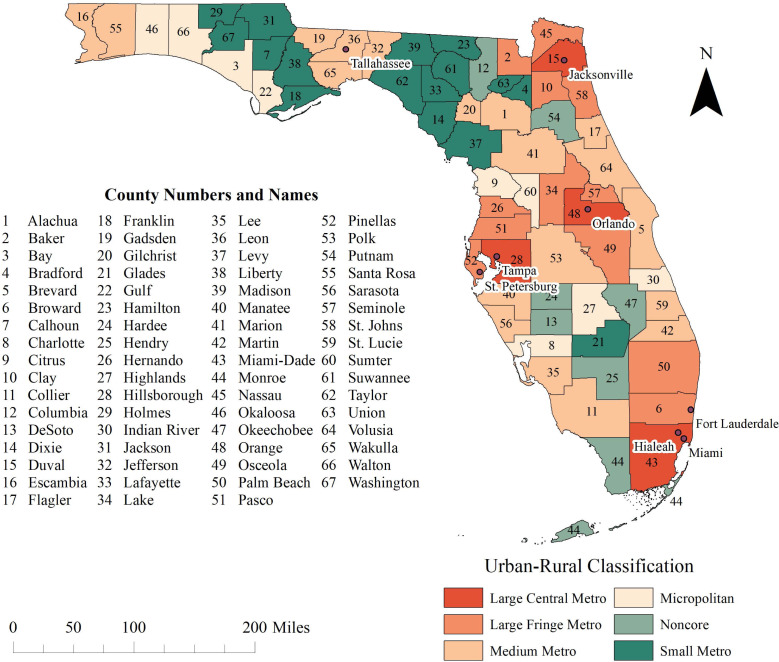
Urban-rural classification and geographic distribution of counties and major cities in the state of Florida, USA.

This ecological study was conducted in Florida, a state that is comprised of 67 counties and includes both rural areas and large urban centers. The state’s estimated population, based on data collected between 2012 and 2016, was 19.9 million, 19.1% of whom were 65 years of age or older (US Census Bureau, 2016a). County populations ranged from 8,285 in rural Liberty County to 2.66 million in Miami-Dade, the most populated county in the state (Fig. 1).

### Data sources and data preparation

The various sources of data used for analysis in the current study are listed in Table 1. Cartographic boundary files for Florida counties were obtained from the US Census Bureau TIGER Geodatabase and the Florida Geographic Data Library (University of Florida GeoPlan Center, 2022; US Census Bureau, 2016b). Behavioral Risk Factor Surveillance System (BRFSS) data for 2013 and 2016 were provided by the Florida Department of Health. State health departments conduct BRFSS surveys, with technical, methodological and financial support from the Centers for Disease Control and Prevention (CDC) (Centers for Disease Control and Prevention, 2019). Every three years, the Florida Department of Health undertakes large sampling, sufficient for obtaining county-level estimates directly from BRFSS data.

**Table 1:** Data sources and variables used in the study of geographic disparities and temporal changes in prevalence of diabetes in Florida.

**Source**	**Data obtained**
County Health Rankings and Roadmaps project	Percent of the population with limited access to healthy foods
Florida Behavioral Risk Factor Surveillance System (BRFSS) (2013, 2016)	Respondent diabetes status (self-reported) Respondent’s county of residence Age of respondent Body mass index of respondent (BMI) Physical activity level of respondent Respondent arthritis status Respondent disability status Respondent health insurance status
Health Resources and Services Administration Area Health Resource Files (2016)	Number of primary care physicians per county
Li et al. (2014)	2010 US Standard Population for age adjustment
National Center for Health Statistics classification scheme (2013)	County rural–urban classification data
United States Census Bureau American Community Survey 5-year estimates (2012–2016 and 2009–2013)	Median household income Percent of the population 16 years and older who are unemployed Percent of the population living below the federal poverty level Percent of the population 25 years and older with less than a high school education Percent of the population 16 years and older who are Hispanic Percent of the population 16 years and older who are non-Hispanic Black Percent of the male population Percent of workers 16 years and older that walked or biked to work Percent of workers 16 years and older that commuted to work for longer than 60 min one way
United States Census Bureau TIGER Geodatabase	County-level cartographic boundary shapefile
United States Census Bureau County Business Patterns (2016)	Number of limited service (fast food) restaurants per county Number of fitness and recreational centers per county

Diabetes status for respondents to the BRFSS survey was based upon self-report that they had been told by a doctor that they had diabetes, not related to pregnancy. Additional variables extracted from the BRFSS database for each respondent included age, body mass index (BMI), leisure time physical activity, arthritis, disability (defined as an activity limitation due to health problems), health insurance coverage, and county of residence. Survey questions with missing responses were excluded from analysis. Data obtained from the BRFSS database were aggregated to the county level prior to analysis using SAS software (SAS Institute, 2016). Age adjustment of diabetes prevalence to the 2010 United States standard population (Li et al., 2014) was performed as follows. First, weighted frequencies of respondents with and without diagnosed diabetes in each of three age groups (18–44, 45–64, and 65 years and older) were computed using the SURVEYFREQ procedure in SAS 9.4 (SAS Institute, 2016). Then, direct age standardization to the 2010 U.S. standard population was performed using age-adjustment weights for these groups (Li et al., 2014).

The 2013 National Center for Health Statistics (NCHS) classification scheme was used for rural–urban designation of Florida counties (Fig. 1) (Ingram & Franco, 2014). In this scheme, metropolitan counties are categorized as either large, medium, or small metro counties. Large metro counties, which have at least 1 million residents, are further subdivided into “central” and “fringe” categories (Ingram & Franco, 2014). Medium metro counties have between 250,000 and 999,000 residents, and small metro counties have under 250,000 inhabitants (Ingram & Franco, 2014). Nonmetropolitan counties are either categorized as micropolitan (with urban cluster populations of between 10,000–49,999 people) or noncore (rural areas that do not qualify as micropolitan) (Ingram & Franco, 2014).

Data on demographic and socioeconomic characteristics of counties, as well as commuting data for county populations, were extracted from the 2012–2016 ACS 5-year estimates (US Census Bureau, 2016a). The following demographic variables were obtained: percentage of the population who were Hispanic, percentage who were non-Hispanic Black, and percentage of males. Socioeconomic variables included median household income, percent unemployment among those 16 years and older, percent of the population with income below the federal poverty level, and percent with less than a high school education among those 25 years and older. Commuting information included percent in each county who walked or biked to work, and percent whose one-way commute to work that was longer than 60 min.

Physician workforce data were obtained from the Health Resources and Services Administration (HRSA) 2016 Area Health Resource Files (Health Resources and Services Administration, 2016). The number of physicians per capita was computed using the number of primary care physicians per county and the total county population. The percent of the population with limited access to healthy foods in each county was obtained from the County Health Rankings and Roadmaps project, which used 2015 data. Criteria used to define limited access to healthy foods include an annual family income of 200% of the federal poverty level or less, and distance from a grocery store (further than 10 miles in rural areas, or one mile in non-rural areas) (University of Wisconsin Population Health Institute, 2019). The number of limited service (fast food) restaurants and fitness or recreational centers in each county in 2016 were obtained from the U.S. Census Bureau County Business Patterns (CBP) data (US Census Bureau, 2016c).

### Descriptive statistics

Descriptive analyses were conducted using SAS 9.4 (SAS Institute, 2016). Continuous variables that were normally distributed, based on results of the Shapiro–Wilk test, were summarized using mean and standard deviation, while median and interquartile ranges were used for non-normally distributed variables.

### Spatial cluster identification and investigation

Tango’s flexible spatial scan statistic (FSSS) was used to identify significant high-prevalence spatial clusters of diabetes using FleXScan software (Tango & Takahashi, 2005). The maximum size for the spatial scanning window was set *a priori* to 15% of the regions in the study area (10 counties) in order to avoid detecting unreasonably large clusters (Tango & Takahashi, 2005). The model was specified as binomial using restricted log-likelihood ratio (LLR). To calculate *p*-values for statistical inference, 999 Monte Carlo replications were used, with a cutoff *p*-value of <0.05 for rejecting the null hypothesis of random spatial distribution of cases. The cluster with the largest value of the restricted LLR was identified as the primary cluster while the rest of the statistically significant clusters were secondary clusters. In order to avoid reporting low-risk clusters, secondary clusters were reported if the prevalence ratio (PR) was greater than or equal to 1.2.

### Investigation of predictors of county-level diabetes prevalence

#### Global model

A global multivariable ordinary least squares regression model was built in SAS 9.4 to identify predictors of county-level diabetes prevalence in 2016 (SAS Institute, 2016). Global models estimate one coefficient for each explanatory variable, averaged over all locations in the study area. Spearman’s rank correlation coefficient was first used to identify highly correlated (— *r*_*s*_— ≥ 0.7) continuous potential predictor variables. To avoid multicollinearity during regression modeling, only one of a pair of highly correlated variables was selected as a potential predictor, based upon biological and statistical considerations. Once potential predictors for consideration in the modeling process were selected, a multivariable model with the outcome of age-adjusted county diabetes prevalence was fit using a two-step process. First, univariable associations between potential predictor variables and county diabetes prevalence were assessed. Variables that had significant univariable associations at a *p*-value of <0.15 were then considered for multivariable modeling. Manual backwards elimination, with a critical *p*-value of 0.05, was performed to fit a multivariable model to the data, with the generalized linear modeling procedure in SAS 9.4 (SAS Institute, 2016). Variance inflation factor (VIF) was used to assess for multicollinearity. Values of VIF ≥10 indicated unacceptably high levels of collinearity between variables in the model (Dohoo, Martin & Stryhn, 2012). If removal of a variable from the model resulted in a change in the estimated regression coefficients of any of the remaining variables of greater than 20%, it was considered as a potential confounder and retained in the model regardless of statistical significance. Residual plots were generated to assess whether assumptions of homoskedasticity and normality of distribution of residuals were met.

#### Local model

Local geographically weighted regression (GWR) models are used to investigate spatial non-stationarity of the relationships between explanatory and dependent variables. These models estimate as many regression coefficients as the number of locations in the study area, and are important for investigating geographically varying associations between dependent and independent variables. GWR4 software was used to investigate if these associations varied by geographical location (Nakaya et al., 2015). Explanatory variables from the final global model for diabetes prevalence were specified as independent variables in the local GWR models. The adaptive bi-square kernel method was used, and the optimal bandwidth was identified using the Golden section search method. Corrected Akaike’s information criterion (AICc) was used to compare model fit. The geographical variability test was used to assess for significant spatial variation in local coefficients for each explanatory variable. Coefficients were considered to have significant spatial variability (non-stationarity) if the difference in AICc reported by the geographic variability test was ≤ −2. Spatial dependence of the residuals of the local GWR model was assessed using Moran’s I with queen contiguity weights, using GeoDa software (Anselin, Syabri & Kho, 2006). Statistical significance was assessed using 999 Monte Carlo replications.

#### Assessment for temporal changes

Two-tailed tests of equality of proportions (or Fisher’s exact tests when appropriate due to sample size) were used to identify significant changes between 2013 and 2016 in diabetes prevalence and county-level predictors from the final multivariable model described above. Since these data were obtained from the BRFSS survey and the American Community Survey, which use random sampling, the values for the two time periods were based on independent samples (Florida Department of Health, 2016; US Census Bureau, 2022). Tests for equality of proportions, with adjustment for multiple comparisons using the Simes method, were performed using R software (R Core Team, 2020).

#### Cartographic displays

County-level data were imported to ArcGIS (ESRI, 2017), which was used to perform all geographic information system (GIS) manipulations and generate maps. Choropleth maps were generated to display age-adjusted diabetes prevalence for 2013 and 2016, using Jenks’ optimization classification scheme (natural breaks) to determine the breakpoints used for display of continuous data (Jenks, 1967). The same intervals used to display 2013 diabetes prevalence were also applied to the 2016 map to enable visual comparison of spatial patterns. Statistically significant changes in county-level diabetes prevalence were also displayed in choropleth maps. In addition, a map was generated to display significant spatial clusters of high diabetes prevalence.

Choropleth maps were also generated to display explanatory variables from the final multivariable regression model as well as statistically significant changes in these characteristics between 2013 and 2016. Local coefficients from explanatory variables that had significant non-stationarity were also imported into ArcGIS and mapped. Local coefficients were displayed for counties with a statistically significant relationship between the explanatory and dependent variable based on the corrected *t*-statistic recommended by da Silva & Fotheringham (2016).

## Results

### Descriptive analyses

There were 36,955 total respondents to the Florida BRFSS survey in 2016. A total of 584 respondents with missing age data were excluded from further analysis; therefore, responses for 36,371 participants were included in the current study. The median age of respondents was 60 years, but ranged from 18 to 99, with an interquartile range of 45 to 71. Self-reported race/ethnicity for the majority of respondents was non-Hispanic White (57.9%), followed by Hispanic (23.4%) and non-Hispanic Black (14.1%).

**Figure 2:**
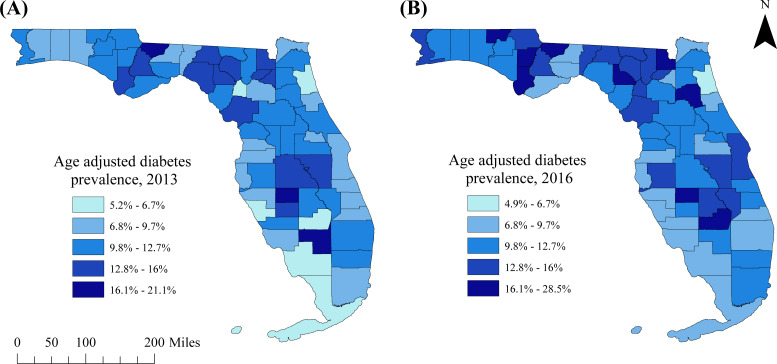
Age-adjusted county-level diabetes prevalence in Florida, (A) 2013 and (B) 2016.

In 2013, the state-wide age-adjusted prevalence of diabetes was 10.1%. In 2016, state-wide diabetes prevalence was slightly higher (10.4%), ranging from 4.9% in St. Johns County to as high as 28.5% in Glades County (Figs. 1 and 2). The rural counties surrounding the Tallahassee area in the panhandle and northern Florida tended to have high prevalence proportions of diabetes. Counties in the inland south-central portion of the state, which were comparatively more rural and less densely populated than those along the Atlantic and Gulf coasts, also had high diabetes prevalence. The major urban centers bordering this region that tended to have comparatively lower prevalence proportions of diabetes included Miami (Miami-Dade County) and Orlando (Orange County). However, Hillsborough County, a large central metro county where Tampa is located, had relatively high diabetes prevalence in 2016.

### Temporal changes in diabetes prevalence

**Figure 3:**
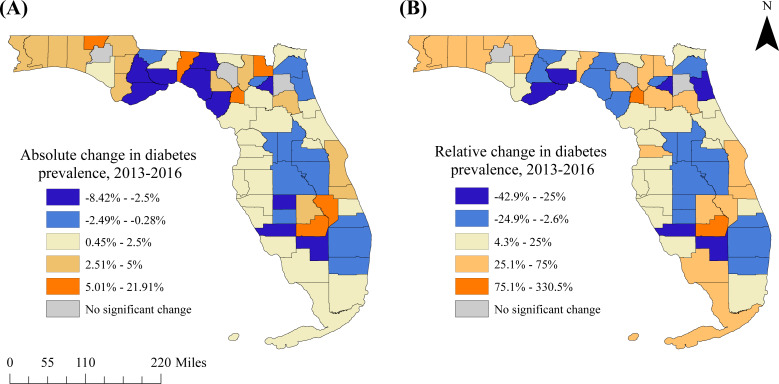
Statistically significant changes in diabetes prevalence in Florida between (A) 2013 and (B) 2016.

There was a small but statistically significant (*p* <  0.0001) increase in state-wide diabetes prevalence between 2013 (10.08%, 95% confidence interval (CI): [10.07%–10.09%]) and 2016 (10.42%, 95% CI: [10.40%–10.43%]). Significant changes in diabetes prevalence between the two time periods were observed in 64 of the 67 counties (Figs. 3A–3B). Only Clay, Suwannee, and Washington counties did not have statistically significant changes between 2013 and 2016 (Figs. 1 and 3). Among the counties with significant changes in diabetes prevalence, 35.9% (23/64) had decreases, while 64.1% (41/64) had increases in diabetes prevalence. Glades County, a rural county in south-central Florida, had the greatest relative increase in diabetes prevalence (21.9%, a relative increase of 330.4%), while Hendry County, the adjacent county to the south, had the greatest relative decrease in diabetes prevalence (8.4%, a relative decrease of 42.9%) (Figs. 1 and 3).

### Spatial clusters of diabetes

**Figure 4:**
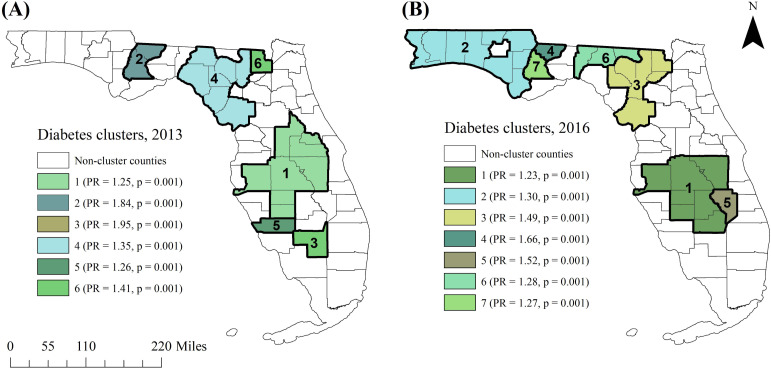
High-prevalence purely spatial clusters of diabetes in Florida, (A) 2013 and (B) 2016.

In 2013, six significant spatial clusters of high diabetes prevalence with PRs ≥1.2 were identified, and seven were identified in 2016 (Table 2, Figs. 4A–4B). The primary cluster in 2013 was located in central Florida, and included both rural counties and the major metropolitan areas of Tampa (Hillsborough County) and Orlando (Orange County). The primary cluster in 2016 included many of the same counties, but shifted south to include more rural counties, and no longer included Orlando (Orange County). The prevalence of diabetes in this cluster was 23% higher than the state prevalence (PR = 1.23, *p* = 0.001). Cluster 5 (PR = 1.52, *p* = 0.001), adjacent to the primary high-prevalence cluster, consisted of a single county, Okeechobee County. The prevalence of diabetes in this county was 15.8% in 2016. With the exception of cluster 5, all secondary diabetes clusters with prevalence ratios ≥1.2 in 2016 were located in north Florida and across the panhandle.

**Table 2:** Purely spatial significant clusters of diabetes in Florida, 2013 and 2016.

**Cluster**	**Population**	**Observed number** **of cases**	**Expected number** **of cases**	**PR** ^a^	***p*** **-value**
***2013***					
1	2,926,257	367,761	294,998	1.25	0.001
2	4,3847	8,168	4,419	1.85	0.001
3	3,5671	7,005	3,596	1.95	0.001
4	173,760	23,578	17,517	1.35	0.001
5	137,671	17,448	13,879	1.26	0.001
6	21,720	3,082	2,190	1.41	0.001
***2016***					
1	1,979,806	254,713	206,276	1.23	0.001
2	819,546	110,750	85,388	1.30	0.001
3	195,164	30,318	20,334	1.49	0.001
4	36,285	6,264	3,781	1.66	0.001
5	40,390	6,395	4,208	1.52	0.001
6	39,474	5,285	4,113	1.28	0.001
7	5,192	686	541	1.27	0.001

**Notes.**

a Prevalence ratio.

In 2016, the two largest secondary diabetes clusters, clusters 2 and 3, were composed of nine and seven counties, respectively. These clusters were located in rural areas in the western panhandle (PR = 1.30, *p* = 0.001) and northern Florida (PR = 1.49, *p* = 0.001). While there was some overlap between high-prevalence diabetes clusters in northern Florida and the eastern panhandle between 2013 and 2016, none of the counties in the western panhandle were part of a high-prevalence cluster in 2013. All the counties in the western panhandle that were included in cluster 2 had statistically significant increases in diabetes prevalence between 2013 and 2016.

### County characteristics and significant predictors of diabetes prevalence

Summary statistics of county characteristics investigated as potential predictors of county diabetes prevalence are displayed in Table 3. The majority of these county characteristics had significant univariable associations with age-adjusted diabetes prevalence (Table 4). The results of the global multivariable model indicated that counties with high diabetes prevalence tended to have high proportions of: non-Hispanic Black population (*p* = 0.020), population with limited access to healthy foods (*p* = 0.018), physically inactive populations (*p* = 0.031), and individuals with arthritis (*p* = 0.032), as well as high unemployment rates (*p* = 0.032) (Table 5). Fitness and recreational facility density was not statistically significant in the global multivariable model (*p* = 0.099), but was a confounder in the associations between county-level diabetes prevalence and levels of unemployment, arthritis, and physical inactivity. Since removal of this variable from the model did not substantially improve model fit to the data (ΔAICc = 0.081) and increased the magnitude of the unemployment, arthritis and physical inactivity variables’ coefficients by 28.5%, 25.4%, and 28.5%, respectively, it was retained in the final global model (Table 5).

**Table 3:** Summary statistics of variables considered as potential predictors of county-level diabetes prevalence in Florida, 2016.

**Predictor variable**	**Mean (SD**^a^)	**Median (IQR**^b^)	**Min. (county)**	**Max. (county)**
Proportion that walk or bike to work^*^	0.021 (0.015)	0.019 (0.013)	0.005 (Washington)	0.111 (Monroe)
Proportion with obesity	0.313 (0.065)	0.306 (0.081)	0.143 (Martin)	0.457 (Union)
Primary care physicians per 1,000 persons^*^	0.548 (0.335)	0.506 (0.429)	0 (Liberty)	2.076 (Alachua)
Proportion with less than high-school education^*^	0.135 (0.062)	0.124 (0.087)	0.044 (St. Johns)	0.326 (Hendry)
Proportion with arthritis	0.282 (0.062)	0.290 (0.075)	0.152 (Wakulla)	0.463 (Glades)
Proportion non-Hispanic Black^*^	0.143 (0.093)	0.117 (0.104)	0.028 (Citrus)	0.536 (Gadsden)
Proportion Hispanic^*^	0.122 (0.120)	0.078 (0.109)	0.017 (Holmes)	0.673 (Miami-Dade)
Median household income (in $10,000)^*^	4.521 (0.838)	4.422 (1.369)	2.981 (Madison)	6.952 (St. Johns)
Proportion with a commute >60 min.^*^	0.083 (0.035)	0.078 (0.049)	0.020 (Hamilton)	0.183 (Bradford)
Proportion physically inactive^*^	0.326 (0.070)	0.311 (0.102)	0.211 (Martin)	0.572 (Dixie)
Fitness & recreational centers per 1,000 persons^*^	0.077 (0.050)	0.073 (0.065)	0 (Calhoun, DeSoto, Dixie, Gadsden, Gilchrist, Glades, Holmes, Jefferson, Lafayette, Union, Washington)	0.246 (Monroe)
Proportion without health insurance coverage^*^	0.169 (0.048)	0.159 (0.054)	0.080 (Sumter)	0.346 (DeSoto)
Proportion with limited access to healthy foods^*^	0.093 (0.057)	0.090 (0.060)	0 (Gilchrist, Wakulla)	0.310 (Glades)
Limited service restaurants per 1,000 persons	0.533 (0.183)	0.531 (0.219)	0 (Liberty)	0.892 (Leon)
Proportion below the federal poverty line^*^	0.111 (0.032)	0.108 (0.036)	0.043 (Sumter)	0.204 (DeSoto)
Proportion reporting a disability	0.236 (0.046)	0.236 (0.064)	0.127 (Miami-Dade)	0.342 (Levy)
Proportion unemployed	0.092 (0.021)	0.087 (0.026)	0.049 (Monroe)	0.150 (Lafayette)
Proportion male^*^	0.507 (0.045)	0.488 (0.055)	0.423 (Okeechobee)	0.607 (Franklin)
NCHS^c^ urban-rural classification^*^	3.746 (1.627)	3 (2)	1	6

**Notes.**

a Standard deviation.

b Interquartile range.

c National Center for Health Statistics.

* Non-normally distributed variables.

**Table 4:** Univariable associations between county characteristics and age-adjusted diabetes prevalence in Florida, 2016.

Predictor variable	Coefficient (95% CI^a^)	*p*-value
Proportion that walk or bike to work	−0.102 (−0.706, 0.502)	0.740
Proportion with obesity	0.302 (0.181, 0.422)	<0.0001
Proportion with overweight/obesity	0.303 (0.180, 0.425)	<0.0001
Primary care physicians per 1,000 persons	−0.049 (−0.073, −0.024)	0.0001
Prop. with less than high-school education	0.285 (0.155, 0.415)	<0.0001
Proportion with arthritis	0.255 (0.121, 0.389)	0.0002
Proportion non-Hispanic Black	0.104 (0.009, 0.199)	0.032
Proportion Hispanic	−0.009 (−0.086, 0.067)	0.808
Median household income (in $10,000)	−0.025 (−0.034, −0.015)	<0.0001
Proportion with a commute >60 min.	0.179 (−0.076, 0.435)	0.169
Proportion physically inactive	0.222 (0.104, 0.341)	0.0002
Fitness & recreational centers per 1,000 persons	−0.438 (−0.589, −0.287)	<0.0001
Proportion without health insurance coverage	0.138 (−0.048, 0.324)	0.145
Proportion with limited access to healthy foods	0.275 (0.131, 0.420)	0.0002
Limited service restaurants per 1,000 persons	−0.082 (−0.128, −0.036)	0.0005
Proportion under the federal poverty line	0.358 (0.090, 0.626)	0.009
Proportion reporting a disability	0.216 (0.026, 0.406)	0.026
Proportion unemployed	1.045 (0.679, 1.411)	<0.0001
Proportion male	−0.016 (−0.217, 0.186)	0.880
NCHS^b^ urban-rural classification	0.009 (0.004, 0.014)	0.0005

**Notes.**

a Confidence interval.

b National Center for Health Statistics.

**Table 5:** Global multivariable regression models predicting county-level age-adjusted diabetes prevalence in Florida, 2016.

**Predictor variable**	**Model 1 (reduced model)**		**Model 2 (full model)**	
	Coefficient (95% CI^a^)	*p*-value	Coefficient (95% CI^a^)	*p*-value
Proportion non-Hispanic Black	0.103 (0.029, 0.177)	0.006	0.088 (0.014, 0.163)	0.020
Proportion unemployed	0.532 (0.173, 0.891)	0.004	0.414 (0.035, 0.793)	0.032
Proportion with limited access to healthy foods	0.144 (0.017, 0.271)	0.026	0.150 (0.025, 0.274)	0.018
Proportion physically inactive	0.140 (0.046, 0.233)	0.003	0.109 (0.010, 0.207)	0.031
Proportion with arthritis	0.165 (0.049, 0.281)	0.005	0.132 (0.011, 0.252)	0.032
Recreational facilities per 1,000 persons	–	–	−0.142 (−0.311, 0.027)	0.099
AICc^b^	−283.970		−284.051	

**Notes.**

a Confidence interval.

b Corrected Akaike’s information criterion.

**Figure 5:**
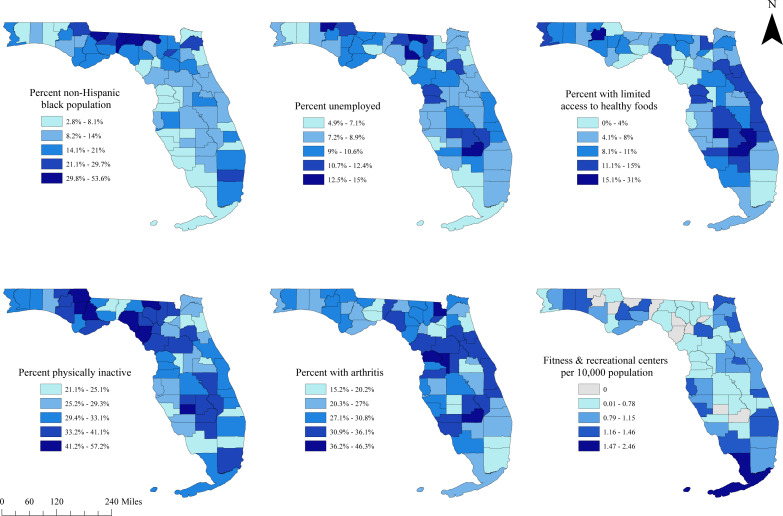
Distribution of predictors of county-level diabetes prevalence in Florida, 2016.

The geographic distributions of the determinants of diabetes geographic disparities identified in the global multivariable model are displayed in Fig. 5. Counties with the highest proportions of non-Hispanic Black residents tended to be located in northern Florida along the border with Georgia, or had large population centers such as Jacksonville, Tampa, and the Miami area. Counties with the highest relative unemployment rates tended to be in rural parts of the state, including inland-south central Florida, northern Florida and the north-central panhandle. Most of the counties with the highest relative proportions of residents with limited access to healthy foods were also located in the inland south-central region, in addition to the central Atlantic coast. Populations in the inland south-central counties also reported relatively high levels of physical inactivity, as did those in the rural counties in the panhandle surrounding Tallahassee. Relatively high arthritis prevalence tended to occur in counties surrounding the Orlando area in central Florida. Rural counties of the eastern panhandle and south-central Florida tended to have low densities of fitness and recreational facilities, while counties with the highest densities of these facilities tended to be along the coasts and closer to metropolitan areas.

The coefficients of several variables exhibited significant non-stationarity; therefore, local geographically weighted regression (GWR) models were fit to the data. Local models were fit for both the full and reduced models to assess the impact of the confounder (fitness and recreational facility density) on the local regression coefficients and their distribution (Table 6). Goodness-of-fit of each GWR model was similar to that of the corresponding global model (ΔAICc (reduced model) = −1.546, ΔAICc (full model) = 1.474), and the optimal bandwidth size in both analyses was 67 counties, which comprised the entirety of the study area. However, in both models, there were significant geographic differences in the strengths of associations between diabetes prevalence and proportion of the population physically inactive, proportion with limited access to healthy foods, proportion unemployed, and proportion with arthritis, as evidenced by the results of the geographical variability test for each variable (Table 6). This implies that the impact and hence importance of these factors varies by geographic location. Thus, some factors may play more important roles in influencing diabetes prevalence in some locations than others. While the strength of associations varied by location for these four variables, the direction of their associations with diabetes prevalence did not change. There was no evidence of spatial dependence in the residuals of either GWR model (Moran’s I (reduced model) = −0.042, *p* = 0.3880; Moran’s I (full model) = −0.030, *p* = 0.4340).

**Table 6:** Geographically weighted regression models predicting county-level age-adjusted diabetes prevalence in Florida, 2016.

**Predictor variable**	**Model 1 (reduced model)**				**Model 2 (full model)**			
	Local coefficients			ΔAICc^b^	Local coefficients			ΔAICc^b^
	Min.	Median (IQR^a^)	Max.		Min.	Median (IQR^a^)	Max.	
Proportion non-Hispanic Black	0.074	0.095 (0.048)	0.152	−1.133	0.058	0.081 (0.053)	0.144	−1.460
Proportion unemployed	0.513	0.531 (0.016)	0.561	−3.286^*^	0.386	0.429 (0.026)	0.462	−4.942^*^
Proportion with limited access to healthy foods	0.068	0.124 (0.109)	0.207	−2.460^*^	0.075	0.140 (0.106)	0.216	−2.232^*^
Proportion physically inactive	0.093	0.129 (0.044)	0.162	−3.228^*^	0.065	0.094 (0.037)	0.131	−3.006^*^
Proportion with arthritis	0.149	0.163 (0.014)	0.191	−9.381^*^	0.117	0.130 (0.010)	0.149	−5.650^*^
Recreational facilities per 1,000 persons	–	–	–	–	−0.169	−0.151 (0.021)	−0.117	1.563
**AICc** ^b^	−285.516				−282.577			

**Notes.**

a Interquartile range.

b Corrected Akaike’s information criterion.

* Indicates significant non-stationarity of local coefficients.

**Figure 6:**
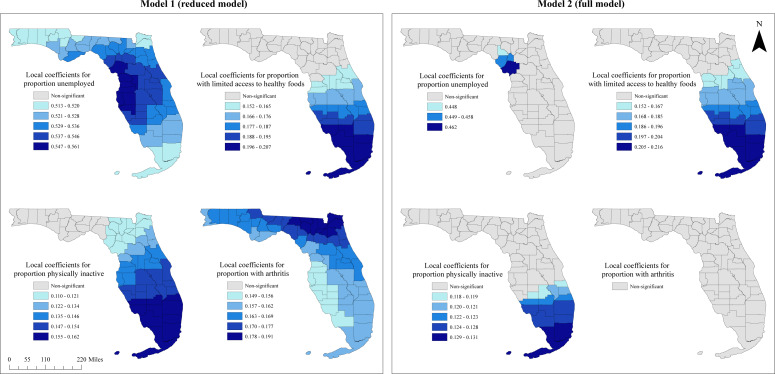
Local coefficients of geographically variable predictors of diabetes prevalence in Florida, 2016.

The number of counties with significant associations between diabetes prevalence and levels of unemployment, physical inactivity, and arthritis decreased substantially when fitness and recreational facility density was included in the full model (Fig. 6). For instance, the association between the unemployment rate and diabetes prevalence in the reduced model was significant in all 67 counties, with the highest local coefficients in west-central Florida, particularly along the Gulf coast. However, in the full model this association was only significant in four counties (Dixie, Gilchrist, Lafayette and Levy counties).

Counties in central and southern Florida had significant associations between diabetes prevalence and proportion of the population with limited access to healthy foods, and the distribution of significant local coefficients, which were highest in the southernmost counties, was similar for the reduced and full models. Similarly, counties with significant local coefficients for proportion of the population reporting physical inactivity extended from the eastern panhandle to southern Florida in the reduced model, with the strongest associations in southern Florida. However, in the full model their distribution was limited to the southern third of the state. The proportion of the population with arthritis was a significant predictor of diabetes prevalence in all 67 counties in the reduced model. This association was strongest in the northeastern portion of the state near Jacksonville and extending to the rural eastern panhandle, and weaker along the Gulf Coast and in southern Florida. However, although the coefficient for this variable was deemed to have significant non-stationarity in the full model based on results of the geographic variability test, local coefficients for arthritis were not statistically significant in any individual counties.

### Changes in county-level characteristics between 2013 and 2016

**Figure 7:**
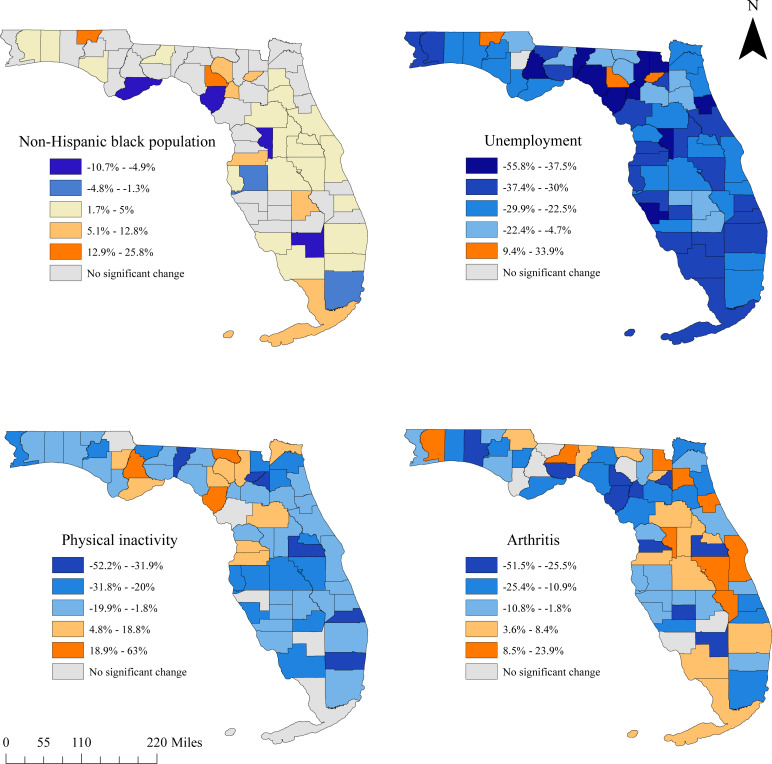
Statistically significant changes in predictors of county-level diabetes prevalence in Florida between 2013 and 2016.

Relative changes in county characteristics with respect to the identified significant determinants of geographic disparities in diabetes prevalence are displayed in Fig. 7. None of the counties had significant changes in fitness and recreational facility density between 2013 and 2016. Significant changes in the proportion of the population that was non-Hispanic Black occurred in about half (50.7%) of the counties in the state, but the magnitude of the changes for many of these counties was less than 5%. The vast majority (95.5%) of the 66 counties with significant changes in unemployment showed decreases in proportion of the unemployed population. Areas with relatively low decreases in the unemployment rate compared to surrounding counties were located in the panhandle and northern Florida near the border with Georgia, and south-central Florida, and tended to have increases in diabetes prevalence between 2013 and 2016.

 Most of the counties with statistically significant increases in the proportion of the population that were physically inactive were located in northern Florida near the eastern panhandle, overlapping with counties with high diabetes prevalence that formed clusters in this region. The majority of counties with statistically significant increases in the proportion of the population with arthritis were located in central Florida between Orlando and Tampa, and tended to be metropolitan areas.

## Discussion

This study investigated geographic disparities in diabetes prevalence in Florida, assessed changes between 2013 and 2016, and identified determinants of these disparities. Study findings are useful for guiding diabetes prevention and control efforts aimed at reducing disease burden and disparities so as to improve population health. Furthermore, the methods used in this study may be applied by other researchers and public health officials in other states to investigate the distribution and determinants of diabetes, or of other chronic conditions.

### Spatial patterns and clusters of diabetes prevalence

Our recent study demonstrated the application of FSSS for the identification of significant spatial clusters, and investigated temporal changes and determinants of pre-diabetes prevalence within Florida during the same time period. In that study, persistent geographic disparities were identified, as were temporal increases in the prevalence of pre-diabetes, indicating that a similar investigation was thus warranted for diabetes (Lord, Roberson & Odoi, 2021). The results of the current study indicate that geographic disparities in diabetes prevalence also continue to exist in Florida, with high-prevalence clusters being identified in 2013 and again in 2016. These findings demonstrate the value of Tango’s flexible spatial scan statistic (FSSS), which improves upon some weaknesses of methods used in other previous studies that have investigated the geographic distribution of diabetes. In addition to eliminating the problem of multiple comparisons, spatial scan statistics avoid pre-selection bias, since the exact location and/or size of suspected clusters are not specified prior to analysis (Kulldorff, 2001). In addition, using Tango’s FSSS enables the detection of irregularly shaped clusters (Tango & Takahashi, 2005). This approach is highly useful for the detection of geographic hotspots, and can be applied to various health outcomes of interest as well as in other states. Counties within the high-prevalence clusters identified in the current study should be prioritized for resource allocation and intervention efforts to mitigate the impacts of diabetes in the population. Continuous monitoring using robust epidemiological techniques is also useful for evaluating the impact of control and intervention programs.

### Predictors of diabetes prevalence

The observed association between diabetes prevalence and the proportion of non-Hispanic Black residents in a county suggests that racial disparities in diabetes prevalence contribute to the observed geographic disparities in the burden of the condition. Racial disparities in diabetes prevalence have been consistently documented in the United States, and the degree of association between race and diabetes is reportedly affected by contextual factors, including socioeconomic conditions (Gaskin et al., 2014; LaVeist et al., 2009; Link & McKinlay, 2009). The proportion of the non-Hispanic Black population was significantly higher in the high-prevalence counties within the diabetes belt in the southeastern US in comparison to other counties, which is consistent with the positive association observed in the current study (Shrestha et al., 2012). This relationship did not exhibit significant spatial variability, consistent with the findings of another study that investigated predictors of diabetes prevalence in counties within and outside of the diabetes belt and found that the percentage of African American residents was a significant determinant regardless of geographic location (Myers et al., 2017).

The association between diabetes prevalence and access to healthy foods observed in this study is also consistent with previous reports that have documented associations between diabetes and characteristics of the food environment (Ahern, Brown & Dukas, 2011; Cunningham et al., 2018). At the individual level, dietary intake is associated with Type 2 diabetes risk (Hu, Van Dam & Liu, 2001; Parillo & Riccardi, 2004), and evidence-based intervention programs such as the Diabetes Prevention Program, which focuses on individual dietary modification and physical activity, have been successful in preventing the progression from pre-diabetes to diabetes (Diabetes Prevention Program Research Group, 2002). Results of the current study provide evidence in support of a relationship between the availability of local food resources and diabetes risk at the county level. The GWR analysis in this study identified spatial non-stationarity in the association between diabetes prevalence and access to healthy foods, suggesting that geographic location influences the relationship between the food environment and diabetes risk. These findings suggest that policies aimed at improving food access could be particularly impactful in counties with relatively high diabetes prevalence in southern Florida, where the relationship between food access and diabetes risk was strongest.

In the current study, levels of unemployment, physical inactivity, and diagnosed arthritis at the county level were positively associated with county-level diabetes prevalence. Notably, however, fitness and recreational facility density, which had significant univariable associations with levels of unemployment (*p* < 0.0001), physical inactivity (*p* = 0.0001), and arthritis (*p* = 0.0037), had a confounding effect on their associations with diabetes prevalence. Inclusion of fitness and recreational facility density in the global model reduced the strengths of these associations, and in the local GWR model, substantially reduced the number of counties where the associations were statistically significant. These findings suggest that the availability of health-promoting community resources may, at least in part, explain the observed relationships between diabetes prevalence and levels of unemployment, physical activity, and arthritis.

Diabetes risk appears to be impacted by the economic context of the living environment in addition to individual economic stability (Andersen et al., 2008; Ludwig et al., 2011). Previous studies in the US have reported that counties with higher unemployment rates tend to have a higher burden of diabetes (Cunningham et al., 2018; Myers et al., 2017). County-level unemployment rates are used to reflect socioeconomic disadvantage and the built environment. Areas with higher levels of unemployment may be characterized by fewer resources that enable health-promoting behaviors such as exercise. Socioeconomic circumstances and characteristics of the built environment may impact diabetes risk by presenting barriers to engaging in recommended physical activity (Booth et al., 2013; Deshpande et al., 2005; Komar-Samardzija et al., 2012). Indeed, our findings suggest that the lack of community resources (in particular, fitness and recreational facilities) may contribute to the diabetes burden in counties with higher unemployment rates, and may also account for geographic disparities in levels of physical inactivity to some extent. The geographically varying strength of the association between diabetes prevalence and physical inactivity exhibited a pattern similar to that of access to healthy foods, with the strongest associations in southern Florida, and no statistically significant relationships in the northern part of the state where several counties are part of the diabetes belt. A previous report identified slightly weaker associations between diabetes status and modifiable risk factors (sedentary lifestyle and obesity) among adults living within diabetes belt counties compared to those living in the rest of the United States (Barker et al., 2011), which was consistent with our findings.

While findings of the global model suggested that the proportion of the population with diagnosed arthritis was a significant determinant of county-level diabetes prevalence, local associations were not statistically significant in any of the individual counties when accounting for the confounding variable, fitness and recreational facility density. At the individual level, arthritis is a common comorbid condition among those with diabetes, and along with obesity, can be a barrier to engaging in physical activity (Booth et al., 2013; Centers for Disease Control and Prevention, 2008; Cheng et al., 2012). The age-standardized prevalence of diagnosed arthritis in the US is higher among adults with lower incomes, overweight or obesity, and those who report physical inactivity (Theis et al., 2021). Thus, while the exact reasons for the findings from our study are not clear, they could reflect associations between built environment resources and proximal risk factors shared by the two conditions.

### Changes in diabetes prevalence between 2013 and 2016

Diabetes prevalence increased both over time and across geographic areas. Since the prevalence estimates were based on self-reports of diagnosed diabetes, it is possible that some of the observed increases may be due to improvements in diagnostic and reporting practices. Indeed, it is worth noting that an increase in total diabetes prevalence in the United States was observed between 1988–1994 and 2005–2010, with a decrease in the proportion of total cases that were undiagnosed (Selvin et al., 2014).

It is possible that the observed changes in some of the county characteristics during the study period contributed to the observed changes in diabetes prevalence. For instance, many of the counties with statistically significant increases in physical inactivity also had increases in diabetes prevalence, suggesting that the observed temporal changes in diabetes prevalence may be attributable to changes in modifiable risk factors in some areas. However, there were some discrepancies in the observed spatial patterns.

The finding that temporal changes in prevalence were observed in many counties indicates that continued monitoring of diabetes and its predictors is warranted to identify sustained changes that could be indicative of emerging trends, in order to guide health programs. While the causes of the observed changes cannot be determined based upon the findings of this study, the fact that county characteristics associated with diabetes prevalence exhibited variable changes across the state highlights the importance of considering local contextual factors when developing public health programming and policies.

### Strengths and limitations

This study was not without limitations. It was conducted retrospectively, using BRFSS survey data, and diabetes status of respondents was self-reported. Thus, diabetes prevalence estimates in this study do not include undiagnosed cases of diabetes and could, to some extent, reflect access to healthcare. Moreover, the BRFSS data used in this study do not distinguish between Type 1 and Type 2 diabetes. However, Type 2 diabetes represents the majority (90–95%) of diabetes cases in the United States (Centers for Disease Control and Prevention, 2017). The above limitations notwithstanding, the present study used robust statistical approaches, applying flexible scan statistics, which are able to identify irregularly shaped clusters and overcome limitations of other cluster detection methods, including problems of multiple testing and pre-selection bias, and local GWR models to investigate geographic disparities and spatially variable determinants of diabetes in Florida. In order to guide targeted health planning and program implementation, ongoing epidemiologic monitoring is essential.

## Conclusions

The findings of this study showed a state-wide increase in diabetes prevalence, as well as increases in many counties in Florida. Geographic disparities in the burden of the condition continue to exist in the state, as evidenced by the identified high-prevalence clusters. These findings are useful for guiding resource allocation geared toward reducing disease burden and reducing disparities. In addition, this study highlights the value of GWR as a tool for understanding the differences in importance of different determinants based on geographic location. The occurrence of spatially varying associations between diabetes prevalence and risk factors implies that a one-size-fits-all approach to disease control is not practical. Thus, needs-based, locally-focused approaches to health planning and service provision are necessary to address disparities and improve population health. Continued monitoring is important for understanding the epidemiology of diabetes and guiding evidence-based control and intervention programs.

##  Supplemental Information

10.7717/peerj.15107/supp-1 Supplemental Information 1 Raw Data

## List of abbreviations

ACS: American Community Survey
AICc: Corrected Akaike’s information criterion
BMI: body mass index
BRFSS: Behavioral Risk Factor Surveillance System
CDC: Centers for Disease Control and Prevention
FSSS: flexible spatial scan statistic
GWR: Geographically Weighted Regression
HRSA: Health Resources and Services Administration
LISA: Local Indicators of Spatial Association
LLR: log-likelihood ratio
NCHS: National Center for Health Statistics
PR: Prevalence Ratio
SAS: Statistical Analysis System
TIGER: Topologically Integrated Geographic Encoding and Referencing
US: United States

## Additional Information and Declarations

### Data Availability

The following information was supplied regarding data availability: The raw data are available in the Supplementary File.
